# Trends in COVID-Related Activity in Sentinel Family Medicine Practices: An Observational Study

**DOI:** 10.3389/ijph.2022.1605361

**Published:** 2023-01-16

**Authors:** Muriel Maeder, Diane Auderset, Bernard Borel, Eric Masserey, Joëlle Schwarz, Yolanda Mueller

**Affiliations:** ^1^ Department of Family Medicine, Centre for Primary Care and Public Health (Unisanté), University of Lausanne, Lausanne, Switzerland; ^2^ Public Health Service, Lausanne, Switzerland

**Keywords:** public health, surveillance, COVID-19, primary care, monitoring, family medicine, sentinel

## Abstract

**Objectives:** During the COVID pandemic, data collected in family medicine were scarce. The COVID-FM project aimed to monitor trends of COVID-related activity in family medicine practices of the canton of Vaud, Switzerland, during the year 2021.

**Methods:** Practitioners were invited to join an *ad hoc* sentinel surveillance system. Online data collection was based on daily activity reports and monthly questionnaires. Participants categorized daily counts of consultations and phone calls into predefined categories. Data were reported and discussed on a weekly basis with public health authorities.

**Results:** On the target of 50 physicians, 37 general physicians from 32 practices finally constituted the COVID-FM sentinel network, contributing to 901 practice-weeks of surveillance in family medicine and 604 in paediatrics. In paediatrics, COVID-related activity corresponded mostly to COVID-19 diagnostic consultations (2911/25990 face-to-face consultations = 11.2%) while in family medicine, other COVID-related topics—such as questions on vaccination—predominated (4143/42221 = 9.8%).

**Conclusion:** COVID-related consultations constituted an important part of primary care practices’ activity in 2021. Monitoring COVID-related activity in primary care provided health authorities with valuable information to guide public health action.

## Introduction

Surveillance systems are the foundation of any communicable disease control strategy, needed for an effective response to a pandemic such as coronavirus disease 19 (COVID-19). For Poon et al. [1], a cohesive response depends on well-integrated primary care and public health systems. In the Swiss federal state, while surveillance of transmissible disease is the responsibility of the national level, organisation of the health system is the responsibility of the cantonal (regional) states. Thus, the Federal Office of Public Health (FOPH) is in charge of the surveillance of transmissible diseases in close collaboration with the cantonal medical officers and healthcare professionals [2, 3]. The Federal surveillance system includes in particular (i) the mandatory reporting of infectious diseases requiring notification, clinical and/or laboratory, and (ii) the sentinel surveillance network—*Sentinella* [4], consisting of about 150–200 family physicians. These systems allow the surveillance of the most frequent communicable diseases and the acquisition of basic knowledge about their spread and their burden in family medicine. Their legal basis is the Federal Act on Control of Communicable Human Diseases, short forms as Epidemics Act or EpidA [5].

During the COVID-19 pandemic, Switzerland has adapted existing epidemiological surveillance systems. COVID-19 was added to the notifiable diseases list on 01st February 2020, and the Sentinella surveillance system adjusted on 19th March 2020, to allow declaration of suspect COVID-19 cases and monitor impact on family medicine practices. In addition, a specific surveillance of admitted COVID-19 cases was set-up in selected Swiss hospitals (CH-SUR) in March 2020 [6].

The cantons being in charge of organizing the health system and executing the measures planned by the Epidemics Act, they can set up additional surveillance systems to these aims. At the beginning of the pandemic, the fear was that it would overwhelm components of the health system, such as hospitals but also nursing homes, transport services or doctor’s on-call services. Thus, in the canton of Vaud, a specific information system for COVID-19—SICOVID [7]—was set up. SICOVID aimed to monitor both the epidemiological situation, and the burden on the health system, providing real-time information to stakeholders responsible for public health decision-making. Management of COVID-19 diagnostic activities was mainly taking place in dedicated testing centres. However, by the end of 2020, it was anticipated that routine structures would take over many COVID-related activities such as testing. To monitor the withdrawal of dedicated centres it was decided to complement the cantonal COVID information system with specific data collection in a panel of family practices. The Cantonal health authority of Vaud (DGS) mandated the Department of Family Medicine (DMF) of Unisanté (Centre for Primary Care and Public Health, University of Lausanne, Lausanne, Switzerland) to develop and implement an *ad hoc* sentinel surveillance system in the Canton of Vaud - the COVID-FM project. Its aim was to monitor trends in COVID-related activities in family medicine and paediatric practices, and to characterise the role of the practices in the management of the pandemic, in particular the deployment of tests and vaccines against SARS-CoV-2. Although primary care sentinel systems were implemented during the COVID-19 pandemic across different European countries [8, 9], these were usually based on pre-existing networks. To our knowledge, no other “*ad hoc*” sentinel network dedicated to monitoring the COVID-19 activities was set up in primary care. In addition, surveillance systems at regional level are rarely described. Thus, this paper intends to describe the regional COVID-FM project and discuss its strengths and limitations.

## Methods

**FIGURE 1:**
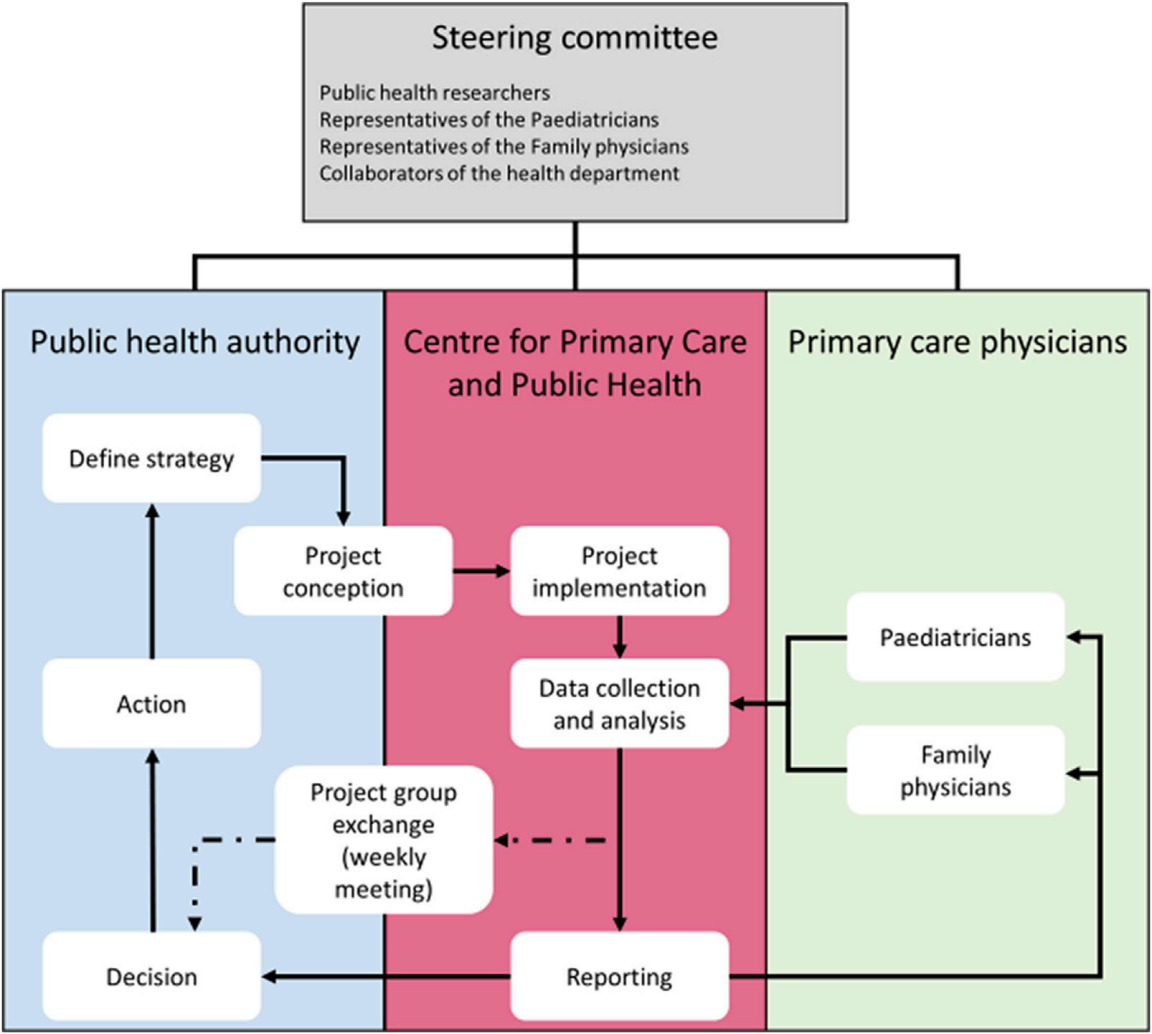
Framework of the COVID-FM project and collaboration between the different partners: on one hand the public health authority and on the other hand the primary care actors (Centre for primary care and public health—Unisanté and the primary care physicians of the COVID-FM network), COVID-FM, canton of Vaud, Switzerland, 2021.

The COVID-FM project was an *ad hoc* sentinel network of family physicians and paediatricians, collecting data prospectively in the canton of Vaud. Data collection was aimed at increasing understanding of the COVID-19 situation in the primary care sector and guiding active decisions in the organization of the pandemic response. Unisanté, the Centre for Primary Care and Public Health, played the interface between cantonal authorities and primary care practices. It managed the different steps in the data processing of the COVID-FM project, from data collection to reporting and dissemination to the Cantonal health authority of Vaud (Figure 1). Reporting was also shared with the participating family practices. The timing of data collection ranged from 22nd March to 31st December 2021.

### Context Information on the Canton of Vaud

The canton of Vaud located in French-speaking Switzerland is the third most populated with over 800,000 inhabitants. The density of physicians in the canton of Vaud for the outpatient sector corresponds to 257 physicians per 100,000 inhabitants. If only doctors holding a specialist title in family medicine were considered, the density is 102 physicians per 100,000 inhabitants, i.e., 39.7% [10–12].

During the first quarter of the year 2021, a partial lockdown situation was in force (shopping facilities for everyday need only), permission of restricted gatherings (<15 people), mandatory mask wearing in public spaces and services, and home-office, if possible. In spring, terraces and cultural spaces were reopened as well as open air popular gatherings (<100 participants) were allowed again. A COVID certificate to access cultural and leisure facilities (e.g., museums, bars) was introduced during the summertime and special rules were set up for “cured or vaccinated” people at the end of the year. All measures were lifted in February 2022. The vaccination campaign started in December 2020 and was in progress during all the year. Access to testing was facilitated throughout the year 2021. Costs of all tests, independently from symptoms, were covered by the Confederation.

### Population

The project population included primary care physicians (general practitioner or paediatrician) practicing in the canton of Vaud, who were not members of the Swiss Sentinella network (nor any of their colleagues in the practice) and worked at least two and a half days a week in the practice. Physicians of the institutional mailing list (N = 969) were invited by email to join the COVID-FM network in January 2021. Participation in the project was on a voluntary basis, with financial compensation for the practice’s involvement in the project. The commitment of each participant was formalized by a convention signed by both parties. While paediatricians represent 19% of primary care physicians in the canton of Vaud [11], their overrepresentation was expected in COVID-FM, and allowed the stratification of the results between family medicine and paediatrics. Based on the responses received, the purposively overrepresentation was effective, without the need to further select neither paediatricians nor family doctors.

### Data Collection

Globally, participants provided information on their practice characteristics, daily practice activity, whether or not related to COVID-19, and on their testing and vaccination activities in the practice.

The practice activities of the participating COVID-FM physicians were recorded using four different questionnaires (see Supplementary Material): the initial questionnaire on practice and physician characteristics, the monthly questionnaire and finally both the daily physician and daily medical assistant (MA) questionnaires. More than one physician could report from a single practice, but it was not required for all physicians from a practice to participate. Medical assistants were asked to categorize patient phone calls per participating physician.

Several key definitions were necessary to allow the construction of the indicators, especially the types of practice activity (both face-to-face or remote medical consultation and call to MA) and the reasons for consultation/call—related or not to COVID-19, and, if related, complying with the national case definition of COVID-suspect in use at the time of data collection [13]. Classification of COVID-related consultations was predefined (see Supplementary Material).

Data from the participating practices were collected and managed using the REDCap® software (Research Electronic Data Capture), hosted by Unisanté [14, 15].

### Data Entry

An online portal was created both to facilitate data entry, and to disseminate reports using the collected data, *via* weekly and monthly reports directly deposited on the platform. Access to the platform and online questionnaires was *via* a unique login (email address and password) for participating physicians. Automatic email reminders were set up to ensure regular data entry.

### Data Protection

Physicians and MA provided aggregated information for practice activities and not for individual patients.

### Statistical Analysis

All data were analysed using Stata statistical software, version 17 (StataCorp. 2021. Stata Statistical Software: Release 17. College Station, TX: StataCorp LLC).

The daily activity data reported by the practices was aggregated by type (face-to-face consultations, remote consultations and telephone calls to MA) and by week, and related to the number of patient-physician contacts (PPC) for the corresponding week—which is equivalent to the sum of face-to-face consultations. Mean weekly COVID-related activity was thus expressed in number of consultations, respectively phone calls, per 1,000 PPC, similarly to influenza surveillance. As the COVID-19-related realities in family medicine and paediatric practices were very different, all analyses were stratified by specialty, and thus presented separately for family physicians and paediatricians. Results were presented in graphical form, with weeks indicated on the horizontal axis, facilitating the temporal visualisation of weekly trends.

### Reporting

Data were processed on a weekly basis and integrated in a weekly report, which included text interpretation of each figure, as well as a separate section of cantonal notification data to put the sentinel data into context. These reports were made available through the COVID-FM portal to health authorities, members of the steering committee and participating physicians. Weekly visioconferences were held between researchers and the public health authority, both to discuss the results and to refine the system, for example for adapting the figures or changing the selection of results presented in the weekly reports.

## Results

### Physician and Practice Characteristics

The target number of physicians for this project was initially of 50, and 46 physicians indicated their interest in participating. In the end, the COVID-FM sentinel network accounted for 37 general physicians who participated regularly throughout the year, from 32 practices. The setting of the medical practices involved in the project was in line with the canton of Vaud reality with 22 practices (26 physicians) in urban area, 8 practices (9 physicians) in suburban area and 2 practices (2 physicians) in rural area setting. All ten districts of the canton were represented.

Physician characteristics are listed in Table 1. Physicians were aged between 39 and 56 years old (median of 47 years old). The median number of employment rate for all physicians was 80% (interquartile range (IQR) of 70-90), without difference between specialties. The median number of physicians in the practices was 2 per practice (IQR 1-2), for a median number of 2 medical assistants (IQR 1-3). In terms of demographic characteristics and activity, gender distribution sentinel physicians was comparable to cantonal statistics, but sentinel physicians were slightly younger [16].

**TABLE 1:** Physician characteristics and organization of practices, COVID-FM, canton of Vaud, Switzerland, 2021.

	Family physicians (N = 22)		Paediatricians (N = 15)		*p*-value	Total (N = 37)	
	Median	IQR	Median	IQR		Median	IQR
Physician age, in years	48.5	(42–56)	47	(39–54)	0.345	47	(42–55)
Physician self-reported sex, n women (%)	8	(36.4%)	9	(60.0%)	0.157	17	(46.0%)
Physician activity rate, in %	80	(70–90)	80	(70–90)	0.740	80	(70–90)
Year of installation	2009	(2006–2015)	2008	(2001–2017)	0.780	2009	(2003–2016)
Number of physicians	1.5	(1–3)	2	(1–2)	0.489	2	(1–2)
Number of medical assistants	1.5	(1–2)	2	(2–3)	0.176	2	(1–3)

IQR, interquartile range.

Practice activity was reported from 22nd March to 31st December 2021, corresponding to 1515 physician-weeks. Per week, practices reported a median of 50 face-to-face consultations (IQR 37-69), 8 remote consultations (IQR 4-15), and 67 phone calls to the medical assistant (MA) (IQR 40-102), details by specialty are reported in Table 2. COVID-19 diagnostic activities represented 4.4% and 11.2% of all reported face-to-face consultations in family medicine and paediatrics, respectively, and other COVID-related consultations represented 9.8% and 2.2%, respectively.

**TABLE 2:** Number and proportion of weeks with activity and weekly number of consultations/calls, by type and by specialty, COVID-FM, canton of Vaud, Switzerland, 2021.

	Family medicine (N = 22)			Paediatrics (N = 15)		
	Face-to-face	Remote	Phone call medical assistant	Face-to-face	Remote	Phone call medical assistant
Number of weeks^a^ (%) with ≥1 consultation/call	743 (82.5%)	646 (71.7%)	789 (87.6%)	523 (85.2%)	425 (69.2%)	478 (77.9%)
Median number of consultations/calls per week (IQR)	54 (38–74)	8 (4–17)	61 (37–95)	46 (35–62)	7 (4–12)	77 (46–122)
Total consultations/calls (represent 100%)	42,221	8,828	54,283	25,990	4,001	42,120
Number of consultations/calls for COVID-19 diagnostic activities (Col %)	1,848 (4.4%)	93 (1.1%)	934 (1.7%)	2,911 (11.2%)	82 (2.0%)	2,121 (5.0%)
Number of other COVID-related consultations/calls (Col %)	4,143 (9.8%)	454 (5.1%)	3,733 (6.9%)	559 (2.2%)	71 (1.8%)	1,352 (3.2%)

^a^ Total practice-weeks for the considered period: 901 in family medicine and 604 in paediatrics.

IQR: interquartile range.

### COVID-19 Diagnostic Activities Over Time

**FIGURE 2:**
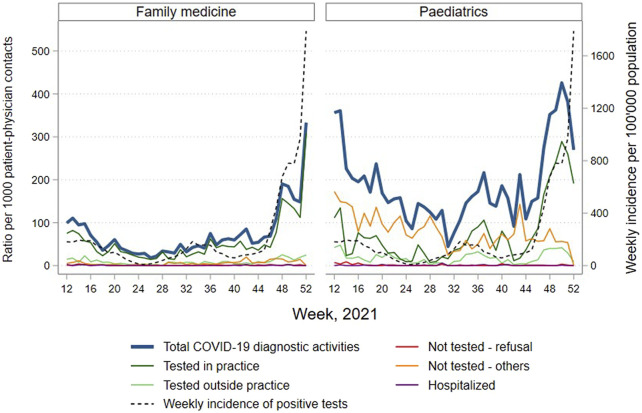
Ratio of number of consultations and calls for COVID-19 diagnostic activities per 1000 patient-physician contacts reported by sentinel family physicians and paediatricians, total and by detailed category, by specialty and by week 2021, COVID-FM, canton of Vaud, Switzerland, 2021. Source for weekly incidence of positive tests: positive SARS-COV-2 tests notified to the Federal Office of Public Health for the canton of Vaud.

The evolution of COVID-19 diagnostic activities in COVID-FM network is depicted in Figure 2. The consultation rate for COVID-19 diagnostic activities was overall higher in paediatrics than in family medicine, probably reflecting the higher incidence of viral infections causing similar symptoms as COVID-19 among children. Almost all patients consulting with suspicious symptoms were tested in family medicine. By contrast, in paediatrics, suspected cases were mostly untested over the whole period, although there was a reversal in the ratio of tested to untested cases in weeks 35–38 and 45–52, corresponding to the 4th and 5th epidemic waves observed based on mandatory reporting. Finally, a higher proportion of suspected cases was carried out outside the office in paediatrics than in family medicine. The rate of hospitalisation of cases seen in the practices remained negligible.

### Consultations and Calls for COVID-19 Excluding COVID-19 Diagnostic Activities

**FIGURE 3:**
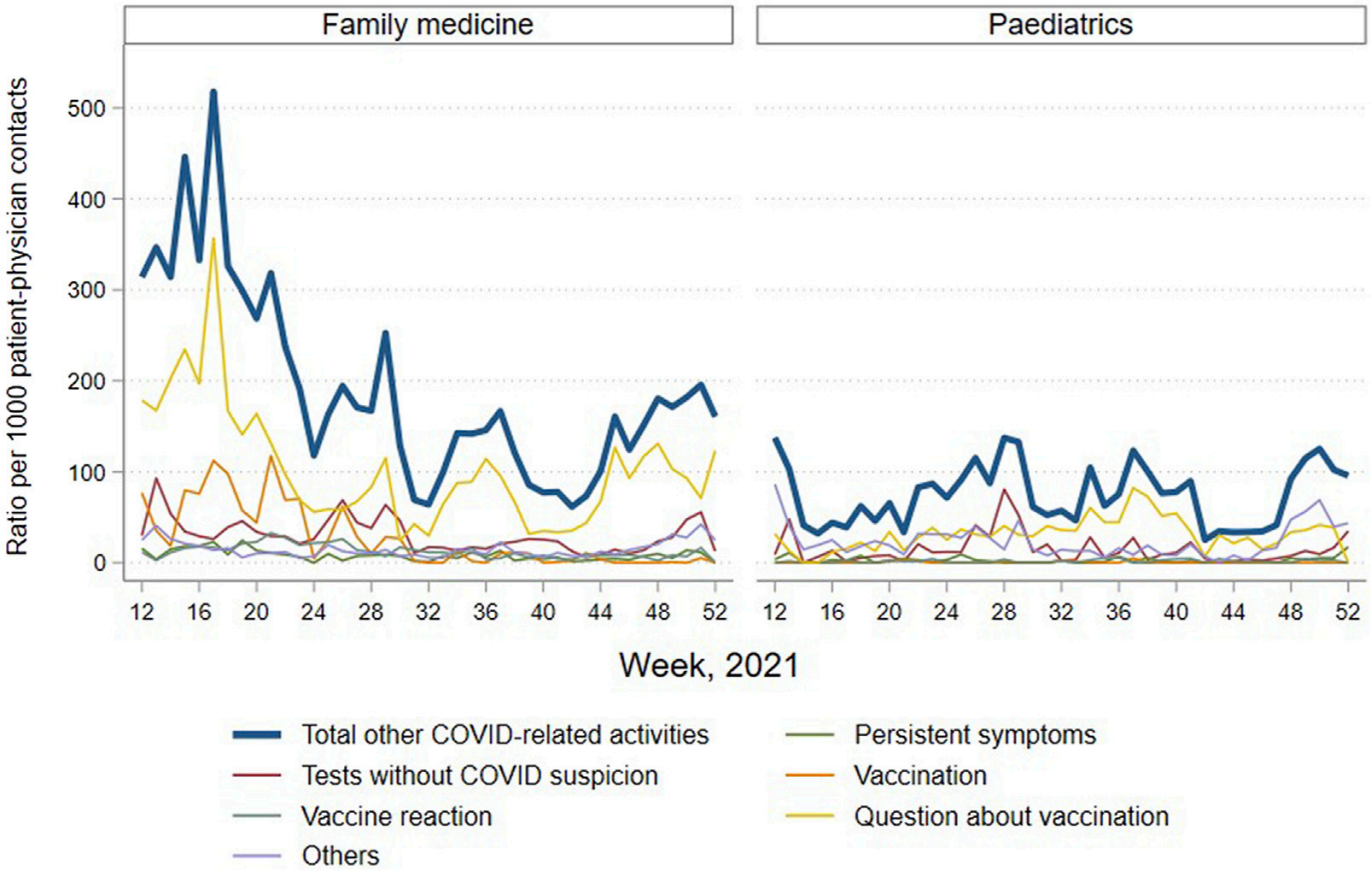
Ratio of new consultations and calls for COVID per 1000 patient-physician contacts, excluding COVID-19 diagnostic activities, reported by sentinel family physicians and paediatricians, total and by category, by specialty and by week 2021, COVID-FM, canton of Vaud, Switzerland, 2021.

In contrast to consultations for COVID-19 diagnostic activities, other consultations and calls related to COVID-19 were reported more frequently in family medicine than in paediatrics, particularly in the spring (weeks 12–23), as presented in Figure 3. The main reason for COVID consultations, outside of diagnosis, was related to questions about vaccination, particularly marked in family medicine. SARS-CoV-2 vaccination was reported in family medicine until the summer. Primary care physicians were no more implicated in vaccination in the second half of the year. The number of tests carried out at the practice outside the COVID-19 diagnostic activities—for example in the context of travel or to obtain a COVID certificate—remained fairly stable at a low level throughout the year, but increased before each school holiday period. The rate of persistent symptoms (possibly indicating long COVID) seen in the practices remained marginal, representing less than 1% of consultations.

### Sampling for SARS-CoV-2 Tests

**FIGURE 4:**
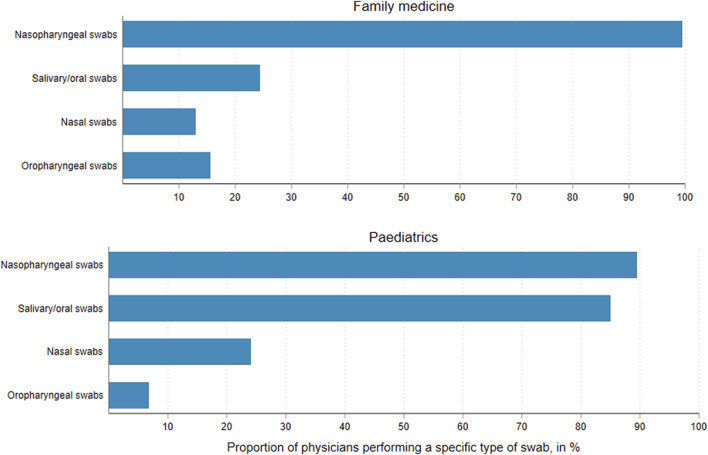
Types of sampling methods used by sentinel family physicians and paediatricians, average over monthly reports, COVID-FM, canton of Vaud, Switzerland, 2021.

Nasopharyngeal (NP) swabs were the type of swabs taken by most COVID-FM physicians. Paediatricians also widely used buccal swabs or salivary samples, favouring methods considered less invasive for younger populations (Figure 4).

## Discussion

Data collected in the COVID-FM sentinel surveillance system has enabled to better understand the practices’ role in the COVID-19 pandemic during the year 2021. The COVID-FM project highlighted the following aspects in the family medicine: (i) the non-essential role of family practices in COVID-19 diagnostic activities; (ii) the important role of counselling by physicians regarding the COVID-19 vaccination; and finally (iii) the limited place of long COVID among all general consultations and calls. In particular, quantitatively, COVID-19 accounted for an important portion of the activity, although it did not appear to have caused major overload. For example, during week 16–17, at the time of the opening of COVID-19 vaccination to a large part of the population, half of all patient contacts involved COVID-related issues. Even if this probably included consultations not initially motivated by COVID-19, COVID-19 turned out to be the main topic, at a time when COVID-19 vaccination was a major concern for patients. In 2020, when the Federal Council ordered health providers to report non-urgent consultations, practices quickly switched to teleconsultations [17]. However, at the time of the COVID-FM project in 2021, activities had mostly returned to normal, as shown by the relatively low number of distance consultations. In fact, the COVID-19 consultation activity partly replaced other reasons for consultation, particularly, in paediatrics where other infectious diseases were less frequent. The burden of COVID-related calls of MA, on the other hand, clearly added to the usual activity. This project also highlighted that the realities in paediatrics and family medicine concerning COVID-19 diverged. Indeed, more consultations for COVID-19 diagnostic activities were observed in paediatrics, while more consultations for other COVID-19-related reasons were reported in family medicine. Almost all patients consulting with suspected symptoms were tested in family medicine. By contrast, the recommendation to systematically test any person presenting COVID suspicion criteria was not applied in paediatrics. Indeed suspected cases in paediatrics were mostly untested over the whole period. The decision to test was weighted by the clinical and epidemiological assessment of paediatricians, probably reflecting the experience of physicians used to distinguishing different viral pathologies on a clinical basis.

In terms of COVID-testing, the COVID-FM project has been useful to emphasize the overall minor role played by medical practices. They have often been considered as a “spare capacity” for their patients, as highlighted by the increase in consultations for tests before school holidays when other testing structures were overwhelmed (hospitals, nursing homes, transport services or doctor’s on-call services). By contrast, a high rate of COVID-related consultations was observed at the time of the roll-out of COVID-19 vaccination. Even if, quantitatively, physicians did not play a major role in vaccinating the population of the canton, these results have also been useful in highlighting the importance they had in counselling patients about this newly available vaccination. During the pandemic, it was important for people to be correctly informed about COVID-19 vaccines [18]. The importance of the counselling role of primary care physicians and the impact it has on primary care activity have been highlighted in other studies [19–21]. This role could be emphasized and better included in preparedness plans. In addition, the COVID-FM project has been useful to emphasize that in relation to all consultations and calls, there were few consultations for long COVID less than 1% and stable throughout the year. Thus, the specific data collection concerning the COVID-19 related activities monitored by the project COVID-FM in medical practices also identified the non-necessity of increased outpatient capacity for dealing with long COVID.

### Strengths and Limitations

The main strength of the COVID-FM project was that it constituted the only observation post for the field of ambulatory medicine in the canton of Vaud in 2021. In terms of the development process, it is important to note the speed of implementation of the system, the functionality of the interface developed, and the interest of the continuous availability of analysis reports to the actors concerned. The COVID-FM platform remains a structure available for other projects or surveys, which potentially can save time during the implementation phases of future projects. The continuous data collection, detailing the reasons for consultation in relation to COVID-19 during the year, has been a particularly useful source of information and has fully fulfilled its role in guiding public health action. In a context where health authorities very much focussed on public hospitals and care institutions, the health ambulatory medicine risked to be overlooked. Surveillance in family medicine and paediatrics is a way for the health authorities to understand the use of care by the population [22]. It is also a way to obtain a comprehensive evaluation of the implementation of the FOPH’s recommendations by physicians.

As mentioned by Rawaf et al. [23], reinforcement of health systems by better collaboration between public health and primary and secondary care to better manage the future health crises is an important lesson of this COVID-19 pandemic. The use of family practice by the population was probably guided by the trust in the physician, and the availability of qualified staff able to answer questions about vaccination or other COVID related questions.

Initially 46 doctors were interested in the COVID-FM project but 9 subsequent withdrawals were recorded, mainly due to the inability of these physicians to find the time necessary to fulfil the requested tasks. The use of a centralized software (electronic patient file), commonly used in other countries (Great Britain or Belgium among others) allowing passive collection of some activities, would certainly be more suitable for this type of project [8, 9]. Another weakness of the COVID-FM project is that, by definition, a sentinel system is not strictly representative of all primary care practices, as recruitment is not based on random sampling. In addition, during the COVID pandemic many symptomatic patients consulted other structures than primary care practices, leading to an under-reporting of both COVID-19 and influenza cases [24]. Besides, in a context were data on test results were almost exhaustively notified, there was no need for the sentinel system to estimate disease incidence. Therefore the role of the sentinel system in pandemic surveillance was actually redefined.

However, being able to rely on an “*ad hoc*” sentinel network at the “meso-level,” corresponding to the regional level where most of the health crisis response was managed, was important. Indeed speediness appeared to be more relevant than strict representativeness during a pandemic.

The motivation of the physicians to be involved in this project was related to their sense of public health duty. The possibility of taking on a leading role in this pandemic that saw them often side-lined was another motivation to participate. The participation in the COVID-FM project was a way for the primary care physicians to get recognition for their essential role during the pandemic. Previous participation in researches for some of them was perhaps also a lever of persuasion. However, the limited number of participating physicians limited possibilities for extrapolation, considering that particular characteristics of one or other practice could have in fact strongly influenced the overall results. Nevertheless, its main use was to monitor trends over time, which allowed to derive interpretable results. For future similar crises, recruitment success could probably be enhanced by a better involvement of primary care organisations in preparedness planning. One could imagine a “watch” sentinel network that could be mobilised “as needed”.

### Conclusion

Monitoring trends in care delivery by primary care practices during the COVID-19 pandemic was a useful contribution for the understanding of the health system response to the pandemic. The implementation of the COVID-FM project allowed us to learn about trends in care delivery by primary care practices from the start and in real time throughout almost 1 year of the COVID pandemic. The feasibility of the project, was demonstrated and can serve as a “proof-of-concept” for other projects.
