# Supplementary material for: Trends in COVID-Related Activity in Sentinel Family Medicine Practices: An Observational Study
Source: Int J Public Health. 2023 Jan 16;67:1605361. doi: 10.3389/ijph.2022.1605361 (PMC9884669; doi:10.3389/ijph.2022.1605361)
Supplement: Supplementary file 1 [file DataSheet1.PDF]

Supplementary material (in French)

Figure S1: Baseline questionnaire, completed only once at the start of the project, providing information on the practice's organisation and the characteristics of the physician participating in the COVID-FM project, COVID-FM, canton of Vaud, Switzerland, 2021

Confidential

Questionnaire initial

Ce questionnaire renseigne les caractéristiques du/ de la médecin membre de COVID-FM et l'organisation du cabinet.

Merci de bien vouloir répondre aux questions suivantes.

Veuillez indiquer votre prénom et votre nom: Prénom _____ Nom _____

Quelle est le code postal (NPA) du cabinet dans lequel vous exercez?	__________________
Quelle est l'adresse e-mail de contact à utiliser dans le cadre du projet COVID-FM?	__________________
Quel est le numéro de téléphone professionnel à utiliser dans le cadre du projet COVID-FM?	__________________
Quel est votre âge?	__________________
Quel est votre genre?	○ Femme<br>○ Homme<br>○ Autre
Quel est votre titre de spécialiste FMH?	○ Médecine interne générale (ou médecine interne, ou médecine générale)<br>○ Pédiatrie<br>○ Médecin praticien·ne<br>○ Autre<br>(Pour rappel: il faut un titre FMH de MIG, pédiatre ou médecin praticien·ne pour être éligible à l'étude COVID-FM)
En quelle année vous êtes-vous installé·e en cabinet?	__________________

Informations sur le cabinet dans lequel vous exercez

Combien de médecin(s) exercent dans votre cabinet, vous y compris, mais sans compter les médecins assistant·e·s	__________________

Nombre de médecins qui exercent dans votre cabinet, vous y compris, mais sans compter les médecins assistant·e·s, par spécialité

	0	1	2	3	4	5	>5
Spécialiste(s) en médecine interne générale	○	○	○	○	○	○	○
Pédiatre(s)	○	○	○	○	○	○	○
Médecin(s) praticien·ne(s)	○	○	○	○	○	○	○

18/02/2021 8:33am projectredcap.org REDCap

Médecin(s) avec une autre spécialité	○	○	○	○	○	○	○

Quel est votre pourcentage d'activité au cabinet? (une demi-journée équivalant à 10%)
____

Quel est le pourcentage total d'activité de l'ensemble des médecins de votre cabinet?
____
(Vous compris)

Quel logiciel informatique utilise votre cabinet pour la saisie des données patient·e·s?
- ○ Mediway
- ○ Achille
- ○ Curamed
- ○ Autre

Merci de spécifier le logiciel informatique
____

Organisation au sein du cabinet

Êtes-vous plusieurs médecins au sein de votre cabinet à participer au réseau COVID-FM?
- ○ Oui
- ○ Non

Veuillez indiquer combien de médecins participent à COVID-FM, vous y compris
____
(Pour rappel, chaque médecin membre doit remplir individuellement un questionnaire initial)

Combien il y a-t-il d'assistant·e·s médical·e·s au sein de votre cabinet
____

Combien d'assistant·e·s médical·e·s sont amené·e·s à répondre aux appels téléphoniques de vos patient·e·s?
____

Combien d'assistant·e·s médical·e·s seront amené·e·s à comptabiliser les appels téléphoniques dans le cadre de COVID-FM?
____

Est-ce que les assistant·e·s médical·e·s arrivent à comptabiliser séparément...

	Oui	Non
...les appels téléphoniques de vos patient·e·s de ceux des patient·e·s d'autres médecins?	○	○
...les appels téléphoniques des patient·e·s des médecins membres de COVID-FM de ceux des patient.e.s des autres médecins ?	○	○

...les appels téléphoniques de chaque médecin membre de COVID-FM (Si non, les appels seront saisis pour l'ensemble des médecins membres)	○	○

Y a-t-il actuellement un·e médecin assistant·e qui reçoit vos patient·e·s ou qui répond au téléphone de vos patient·e·s?	○ Oui<br>○ Non
Si oui, est-ce-que ce/cette médecin assistante serait en mesure de rapporter les consultations pour COVID-FM?	○ Oui<br>○ Non
Y a-t-il actuellement un·e ou des infirmiers·ères qui reçoivent vos patient·e·s ou qui répondent aux téléphones de vos patient·e·s?	○ Oui, une ou un infimier<br>○ Oui, plusieurs infirmiers·ères<br>○ Non, aucune infirmier·ère
Si oui, combien d'infirmières et/ou d'infirmiers en tout?	____________________
Est-ce que ces infirmiers·ères font des consultations ayant pour motif la COVID?	○ Oui<br>○ Non
Si oui, est-ce-que ces infirmiers·ères seraient en mesure de rapporter les consultations pour COVID-FM?	○ Oui<br>○ Non
Est-ce que votre cabinet fait partie du projet pilote MOCCA?	○ Oui<br>○ Non

Figure S2: Monthly questionnaire, providing information on general screening and vaccination activity, specific COVID-related cases (re-infection and long COVID cases), as well as any organisational changes affecting the data collection, COVID-FM, canton of Vaud, Switzerland, 2021

Confidential

Questionnaire mensuel

Ce questionnaire renseigne les activités du cabinet liées à la COVID. Il se réfère à la période écoulée depuis le dernier questionnaire, ou au cours du dernier mois s'il s'agit du premier remplissage

Merci de bien vouloir répondre aux questions suivantes.

Informations sur les tests pour SARS-CoV-2 au sein du cabinet

Votre cabinet réalise-t-il des prélèvements pour recherche de SARS-CoV-2?	○ Oui<br>○ Non
Si ce n'est pas le cas, vous référerez les patient·e·s à:	☐ Un centre de test<br>☐ Un laboratoire<br>☐ Un autre cabinet<br>☐ Une pharmacie<br>☐ Un hôpital<br>(plusieurs réponses possibles)
Si oui, votre cabinet fait-il partie des centres dédiés mentionnés sur le site de l'Etat de Vaud?	○ Oui<br>○ Non
Quels types de prélèvements?	☐ Des frottis nasopharyngés<br>☐ Des prélèvements salivaires<br>☐ Des frottis buccaux<br>☐ Des frottis nasaux<br>☐ Des frottis oropharyngés<br>☐ Autres<br>(plusieurs réponses possibles)
Si autres, veuillez spécifier:	__________________________________
Dans quel cadre?	☐ Avec consultation médicale<br>☐ Sans consultation médicale<br>☐ Chez des patients symptomatiques<br>☐ Chez des patients asymptomatiques<br>(plusieurs réponses possibles)
Qui effectue les prélèvements?	☐ Un·e assistant·e médical·e<br>☐ Vous-même<br>☐ D'autres médecins<br>☐ Autre profession (personnel infirmier, stagiaire, etc.)<br>(plusieurs réponses possibles)
Votre cabinet réalise-t-il des tests rapides antigéniques du SARS-CoV-2?	○ Oui<br>○ Non
Qui effectue les tests rapides?	☐ Un·e assistant·e médical·e<br>☐ Vous-même<br>☐ D'autres médecins<br>☐ Autre profession (personnel infirmier, stagiaire, etc.)<br>(plusieurs réponses possibles)
Avez-vous un identifiant COFAST?	○ Oui<br>○ Non

Utilisez-vous un identifiant personnel ou partagez-vous un identifiant entre plusieurs médecins?	◯ Identifiant personnel<br>◯ Identifiant partagé
Est-ce qu'il arrive que vous demandiez ou réalisiez des tests SARS-CoV-2 pour d'autres personnes que vos patient·e·s ?	☐ Oui, pour des patient·e·s des mes collègues<br>☐ Oui, pour des patient·e·s non suivi·e·s habituellement au cabinet<br>☐ Oui, pour des membres de la famille de mes patient·e·s<br>☐ Non, je ne teste que mes patient·e·s<br>(plusieurs réponses possibles)

Vaccination COVID-19

Est-ce que vous vaccinez contre la COVID-19 au cabinet?	◯ Oui<br>◯ Non
Si non, souhaitez-vous vacciner contre la COVID-19 ?	◯ Oui<br>◯ Non
Si oui, qui vaccine vos patient·e·s?	☐ Un·e assistant·e médical·e<br>☐ Vous-même<br>☐ D'autres médecins<br>☐ Autre profession (personnel infirmier, stagiaire, etc.)<br>(plusieurs réponses possibles)

Est-ce qu'il vous arrive...

	Oui	Non
...de vacciner des patient·e·s de votre/vos collègues?	◯	◯
...de vacciner des personnes non-suivies habituellement au cabinet?	◯	◯

Avez-vous un identifiant VACOVID?	◯ Oui<br>◯ Non
Utilisez-vous un identifiant personnel ou partagez-vous un identifiant entre plusieurs médecins?	◯ Identifiant personnel<br>◯ Identifiant partagé
Quel type de vaccin avez-vous reçu en cabinet jusqu'à présent?	☐ AstraZeneca<br>☐ Moderna<br>☐ Pfizer/BioNTech<br>☐ Curevac<br>☐ Novavax<br>(plusieurs réponses possibles)
Combien de doses de AstraZeneca votre cabinet a-t-il reçues jusqu'à présent?	____________<br>(depuis le début de l'année)

Combien de doses de Moderna votre cabinet a-t-il reçues jusqu'à présent?	______________________ (depuis le début de l'année)
Combien de doses de Pfizer/BioNTech votre cabinet a-t-il reçues jusqu'à présent?	______________________ (depuis le début de l'année)
Combien de doses de Curevac votre cabinet a-t-il reçues jusqu'à présent?	______________________ (depuis le début de l'année)
Combien de doses de Novavax votre cabinet a-t-il reçues jusqu'à présent?	______________________ (depuis le début de l'année)
Au cabinet, est-ce qu'il vous arrive de manquer de vaccins COVID-19?	◯ Oui<br>◯ Non<br>◯ En partie (l'offre est supérieure à la demande ou il manque l'un des types de vaccin)

Cas particuliers liés à la COVID

Depuis le questionnaire mensuel précédent (ou au cours du dernier mois s'il s'agit du premier remplissage), avez-vous traité des patient·e·s atteint·e·s du COVID-19 avec une possible réinfection*?<br>* deux détections positives au SARS-CoV-2 à des intervalles de trois mois ou plus chez le/la même patiente	◯ Oui<br>◯ Non<br>(Le questionnaire journalier ne comptabilise que les suspicions COVID. Les cas de confirmation de l'infection SARS-CoV-2 chez des patient·e·s déjà infecté·e·s par le passé doivent être annoncés ici)
Si oui, combien?	______________________ (Rappel: depuis le questionnaire mensuel précédent (ou au cours du dernier mois s'il s'agit du premier remplissage))
Depuis le questionnaire mensuel précédent (ou au cours du dernier mois s'il s'agit du premier remplissage), avez-vous traité des patient·e·s atteint·e·s du COVID-19 après avoir reçu un vaccin COVID?	◯ Oui<br>◯ Non<br>(Le questionnaire journalier ne comptabilise que les suspicions COVID. Les cas de confirmation de l'infection SARS-CoV-2 chez des patient·e·s déjà vacciné·e·s doivent être annoncés ici)
Si oui, combien?	______________________ (Rappel: depuis le questionnaire mensuel précédent (ou au cours du dernier mois s'il s'agit du premier remplissage))

Figure S3: Daily physician questionnaire providing information on COVID-19-related activities such as face-to-face or teleconsultation, new suspicion of COVID-19 or other COVID-19 related activity.

Feuille de comptage journalière MEDECIN

Si plusieurs feuilles Médecin ont été remplies au cours de la journée, merci de reporter ici le total des comptages

---

Type de consultations

En présentiel À distance
A) Nombre total de consultations facturées ______ ______
B) Nombre total de consultations remplissant les critères d'une nouvelle suspicion COVID-19 ______ ______
B1... avec patient·e référé·e hors cabinet ______ ______
B2... avec patient·e adressé·e pour un test en cabinet ______ ______
B3... non-testé·e pour cause de refus ______ ______
B4... non-testé·e pour autre raison ______ ______
BA... avec patient·e référé·e à l'hôpital ______ ______
BB... chez un·e patient·e ayant déjà eu une infection SARS-CoV-2 documentée par le passé ______ ______
BC... chez un·e patient·e vacciné·e contre le SARS-COV-2 ______ ______
C) Nombre total de consultations liées à la COVID mais ne remplissant pas les critères d'une nouvelle suspicion COVID-19 ______ ______
C1... adressé·e pour un test au cabinet ______ ______
C2... avec des symptômes persistants après l'épisode initial ______ ______
C2A... dont patient·e·s avec des symptômes persistants ≥ 28 jours ______ ______
C3... avec une réaction à la vaccination SARS-CoV-2 ______ ______
C3A... ... dont nombre de déclarations de pharmacovigilance à Swissmedic ______ ______
C4... avec une question sur la vaccination SARS-CoV-2 ______ ______
C5... pour être vacciné·e contre SARS-CoV-2 ______
C7... Nombre de patients pris en charge en lien avec la COVID-19 ne rentrant pas dans les catégories ci-dessus ______ ______
D) Nombre total de sérologies demandées ______ ______
Nombre de sérologies, par motif:
D1 Dispense de vaccination ______ ______
D2 Confirmation d'une ancienne infection ______ ______
D3 Contrôle post-vaccination ______ ______
D4 Avant un voyage ______ ______
D5 Autre ______ ______

Date du questionnaire ______________________________

Pas de données
○ Vrai
○ Faux

____

Figure S4: Daily medical assistant questionnaire providing information on COVID-19-related phone calls from patients.

Page 1

Feuille de comptage journalière ASSISTANT·E MEDICAL·E

Pour rappel: ne comptabilisez pas les appels transférés à la ou au médecin et additionnez les appels de toutes les feuilles de comptage ASSISTANT·E MEDICAL·E de la journée si plusieurs ont été remplies

---

Comptage des appels

Total des appels
A) Nombre total de téléphones (avec ou sans COVID) ______
B) Nombre total de patient·e·s ayant rempli les critères d'une nouvelle suspicion COVID-19, dont... ______
B1... Nombre référés pour un test hors cabinet ______
B2... Nombre de patient·es pour lesquels un RDV a été pris pour un test en cabinet ______
B3... Nombre de patient·es non-testé·es pour cause de refus ______
B4... Nombre de patient·es non-testé·e pour autre raison ______
C) Nombre total d'appels de patient·e·s liés à la COVID-19 mais ne remplissant pas les critères d'une nouvelle suspicion COVID, dont... ______
C1... Nombre de patient·es pour lesquels un RDV a été pris pour un test en cabinet ______
C2... Nombre d'appels pour symptômes persistants après l'épisode initial ______
C3... Nombre d'appels pour réactions à la vaccination ______
C4... Nombre d'appels pour questions sur la vaccination ______
C6... Nombre de patients pris en charge en lien avec la COVID-19 ne rentrant pas dans les catégories ci-dessus ______

Date du questionnaire ______________________________

Pas de données
◯ Vrai
◯ Faux

C5... Nombre de patient·e·s qui téléphonent suite à une notification de l'app SwissCovid ______________________________

09/03/2022 1:17pm

projectredcap.org REDCap

Figure S5: Categorization of face-to-face and teleconsultations by physician

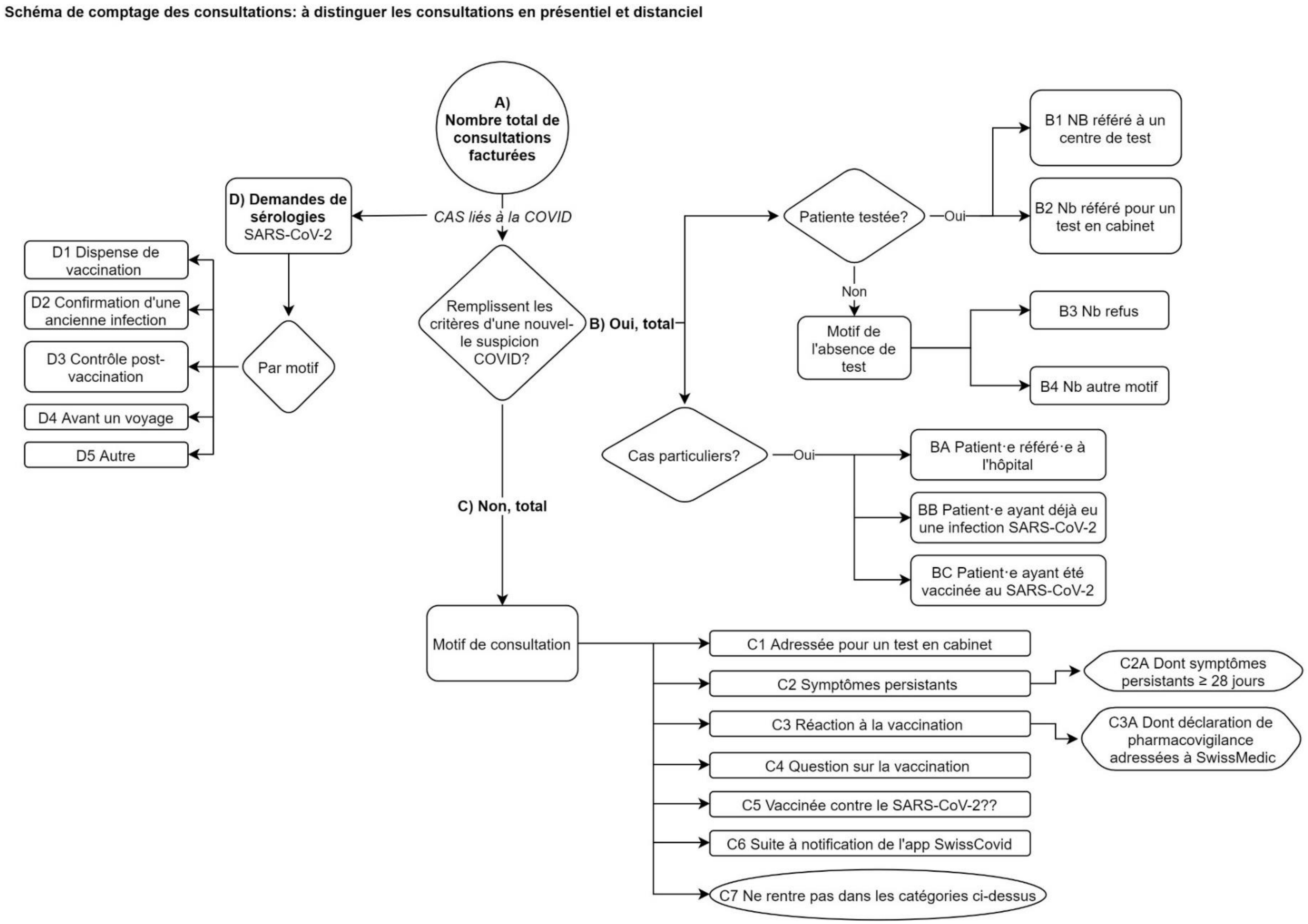

Figure S6: Categorization of phone calls from patients to the medical assistant

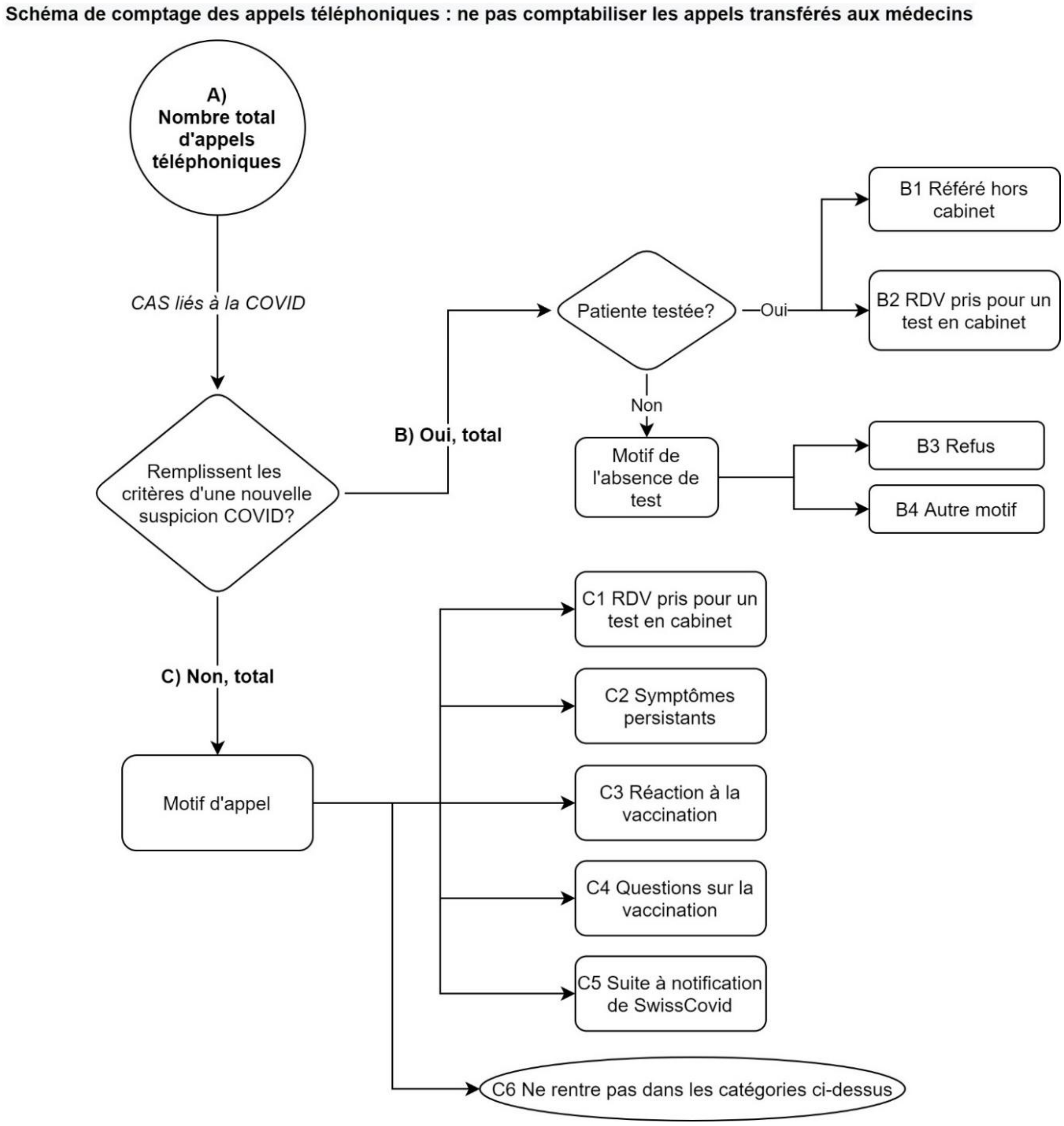

Figure S7: Tally sheet for daily physician activity, designed to easily and systematically record consultations or telephone calls according to predefined categories

unisanté

Centre universitaire de médecine générale et santé publique · Lausanne

Département Médecine de famille

COVID-FM: Feuille de comptage MEDECIN

Des instructions se trouvent au verso

dmf.covidfm@unisante.ch
https://www.unisante.ch/covid-fm

COVID-FM: Feuille de comptage journalière MEDECIN

Date __ __ / __ __ / 2021		Initiales __ __			
Consultations		**En présentiel** (y compris visites à domicile)		**À distance** (tél., mail, visio)	
		Comptage	*Total*	Comptage	*Total*
A	**Nombre total de consultations facturées** (avec ou sans COVID-19)				
B	Nombre de consultations remplissant les critères cliniques OFSP **d'une nouvelle suspicion COVID-19***				
B1	… avec patient·e référé·e hors cabinet				
B2	…avec patient·e adressé·e pour un test SARS CoV-2 au cabinet				
B3	… non-testé·e pour cause de refus				
B4	… non-testé·e pour autre raison				
BA	… avec patient·e référé·e à l'hôpital				
BB	… chez un·e patient·e ayant déjà eu une infection SARS-CoV-2 documentée par le passé				
BC	…chez un·e patient·e vacciné·e contre le SARS-CoV-2				
C	Nombre total de consultations <u>liées à la COVID</u> mais **qui ne remplissent pas les critères d'une nouvelle suspicion de COVID-19**, dont patient·e·s:				
C1	… **adressé·e pour un test** SARS CoV-2 au cabinet				
C2	… **avec des symptômes persistants** après l'épisode initial				
C2 A	… dont patient·e·s avec des symptômes persistants **≥ 28 jours**				
C3	…**avec une réaction à la vaccination** SARS-CoV-2				
C3 A	… dont nombre de déclarations de pharmacovigilance post-vaccination adressées à Swissmedic				
C4	…avec une **question sur la vaccination** SARS-CoV-2				
C5	… **pour être vacciné·e** contre SARS-CoV-2				
C7	… Nombre de patient·e·s pris·es en charge **en lien avec la COVID-19 ne rentrant pas dans les catégories ci-dessus**				
D	**Nombre total de sérologies SARS-COV-2 demandées**				
	Nombre de sérologies par motif :				
D1	- dispense de vaccination				
D2	- confirmation d'une ancienne infection (hors discussion d'une indication vaccinale)				
D3	- contrôle post-vaccination				
D4	- avant un voyage				
D5	- autre				

20.04.2021

Données saisies ☐

unisanté

Centre universitaire de médecine générale
et santé publique · Lausanne

Département Médecine de famille

COVID-FM: Feuille de comptage MEDECIN

Des instructions se trouvent au verso

dmf.covidfm@unisante.ch
https://www.unisante.ch/covid-fm

Instructions :

Les cellules de comptage sont pensées comme une aide à la récolte d'information, étant faciles à noter au fur et à mesure, avant de faire le total en fin de journée. Si le nombre total de consultations ou un autre indicateur peut être extrait du logiciel du cabinet ou de l'agenda papier, notez simplement le total (ligne A).

- Comptabilisez **chaque consultation facturée**, qu'elle ait lieu en présentiel (colonne de gauche) ou à distance (colonne de droite) ;
  - Une consultation pour un autre motif (p.ex. contrôle du diabète) au cours de laquelle la COVID-19 a été évoquée ne doit pas être comptabilisée
- Pour **chaque consultation en présentiel ou à distance ayant comme motif la COVID-19**, il devrait y avoir au moins une coche soit dans la ligne B (suspicion COVID-19), ou la ligne C (autre COVID-19)
  - Si vous avez coché « B », il devrait y avoir aussi une coche dans B1, B2, B3, B4
  - En plus, selon le cas de figure, cochez BA, BB ou BC (plusieurs réponses possibles)
  - Si vous avez coché « C », il devrait y avoir aussi une coche dans C1, C2, C3, C4, C5 ou C7
    - En plus, si vous cochez C2, regardez s'il est nécessaire de cocher C2A ; idem concernant C3 et C3A
  - S'il y a plusieurs points évoqués lors de la consultation (par exemple « j'ai des symptômes mais j'ai aussi une question sur la vaccination »), notez le motif principal selon vous.
- Les **demandes de sérologies** sont comptabilisées indépendamment des motifs de consultations. Par exemple, si un·e patient·e vient pour un suivi du diabète et que vous décidez de faire une sérologie, cochez D puis distinguez selon le motif D1, D2, D3, D4 ou D5
- Les demandes de sérologies sont comptabilisées le jour où vous en faites la demande, pas lors du rendez-vous pour la prise de sang.
- Les téléphones gérés uniquement par l'assistant·e médical·e sont comptabilisés dans une feuille de comptage séparée, rempli par l'assistant·e ;
- Un·e patient·e pris·e en charge par téléphone, puis en présentiel, est noté·e deux fois.
- Comptabilisez également les consultations/appels faits pour des patient·e·s habituellement suivi·e·s par vos collègues.

Les lettres indiquées en marge facilitent l'entrée régulières des données dans le portail en ligne du projet COVID-FM www.covidfm.unisante.ch

Exemple de comptage :

Date 08 / 02 / 2021		Initiales DA			
	Consultations	**En présentiel** (y compris visites à domicile)		**À distance** (tél., mail, visio)	
		Comptage	***Total***	Comptage	***Total***
a	**Nombre total de consultations** (avec ou sans COVID)	卌 卌 卌 卌 II	22	卌 卌 IIII	14
	Nombre de consultations ayant comme **motif** ...				
b	... **une suspicion COVID**	卌 II	7	II	2
C	... avec patient·e référé·e à un centre de test	I	1		
D	...avec patient·e adressé·e pour un test au cabinet	III	3	I	1
E	... non-testé par cause de refus				
F	... non-testé pour autre raison				

* Lien vers **Critères cliniques de suspicion** COVID-19 de l'OFSP: https://www.bag.admin.ch/dam/bag/fr/dokumente/mt/msvs/covid-19-verdachts-meldekriterien.pdf.download.pdf/Criteres_de_suspicion_de_prelevement_d-echantillons_et_de_declaration.pdf

20.04.2021

Données saisies ☐

Figure S8: Tally sheet for daily medical assistant activity, designed to easily and systematically record telephone calls according to predefined categories

unisanté

Centre universitaire de médecine générale et santé publique · Lausanne

Département Médecine de famille

COVID-FM: Feuille de comptage ASSISTANT·E MEDICAL·E

Des instructions se trouvent au verso

dmf.covidfm@unisante.ch
https://www.unisante.ch/covid-fm

COVID-FM: Feuille de comptage journalière ASSISTANT·E MEDICAL·E

RAPPEL : **Ne comptabilisez pas les appels transférés à la ou au médecin**

Date ___ ___ / ___ ___ / 2021		Initiales / Identifiant poste de travail ___ ___ ___	
**Appels téléphoniques**		*Comptage*	*Total*
A	**Nombre total de téléphones non référés** au médecin (avec ou sans COVID-19)		
B	**Nombre total de patient·e·s ayant rempli les critères de l'OFSP d'une nouvelle suspicion de** COVID-19*, dont …		
B1	… Nombre référés pour un test hors cabinet		
B2	… Nombre de patient·e·s pour lesquels un rendez-vous a été pris pour un test en cabinet		
B3	… Nombre de patient·e·s qui ne veulent pas être testé·e·s		
B4	… Nombre de patient·e·s non testé·e·s pour autre raison		
C	**Nombre total d'appels téléphoniques** liés à la COVID **mais qui ne remplissent pas les critères d'une nouvelle suspicion de COVID-19**, dont :		
C1	… Nombre de patient·e·s pour lesquels un rendez-vous a été pris pour un test en cabinet		
C2	… Nombre d'appels pour symptômes persistants après l'épisode initial		
C3	… Nombre d'appels pour réactions à la vaccination		
C4	… Nombre d'appels pour questions sur la vaccination		
C6	…Nombre de patient·e·s pris en charge **en lien avec la COVID-19 ne rentrant pas dans les catégories ci-dessus**		

20.04.2021

Données saisies ☐

unisanté
Centre universitaire de médecine générale et santé publique · Lausanne
Département Médecine de famille

COVID-FM: Feuille de comptage ASSISTANT·E MEDICAL·E

Des instructions se trouvent au verso

dmf.covidfm@unisante.ch
https://www.unisante.ch/covid-fm

Instructions :

Les cellules de comptage sont pensées comme une aide à la récolte d'information, étant facile à cocher au fur et à mesure, avant de faire le total en fin de journée dans la dernière colonne (total).

Ne comptabilisez pas les appels transférés à la ou au médecin, car ceux-ci sont comptabilisés directement par elle ou lui. Nous vous conseillons donc de compléter la feuille en fin de téléphone, lorsque vous êtes sûr·e que l'appel ne sera pas transféré.

Les lettres indiquées en marge facilitent la compréhension des liens entre les questions, ainsi que l'entrée régulières des données dans le portail en ligne du projet COVID-FM www.covidfm.unisante.ch

- **Pour chaque appel non transféré, il devrait y avoir de toute façon une coche pour la ligne A** (COVID-19 et non-COVID-19), **puis, si le cas est lié à la COVID, au moins une à la ligne** B (nouvelle suspicion COVID-19), **ou à la ligne** C (autre COVID-19)
  - Si vous avez coché « B », il devrait y avoir aussi une coche dans B1, B2, B3 ou B4,
  - Si vous avez coché « C », il devrait y avoir aussi une coche dans C1, C2, C3, C4, ou C6
  - S'il y a plusieurs points évoqués lors du téléphone (par exemple « j'ai des symptômes, mais j'ai aussi une question sur la vaccination »), notez le motif principal de l'appel selon vous.

Exemple de comptage :

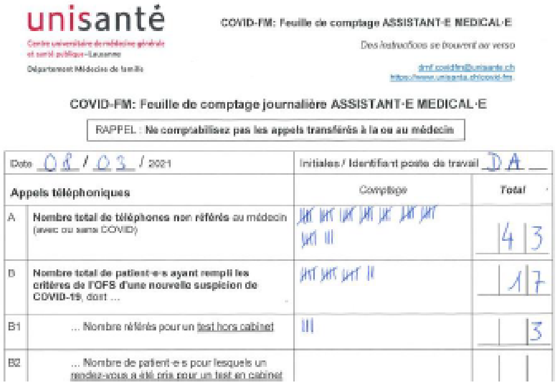
unisanté
Centre universitaire de médecine générale et santé publique · Lausanne
Département Médecine de famille

COVID-FM: Feuille de comptage ASSISTANT·E MEDICAL·E

Des instructions se trouvent au verso

dmf.covidfm@unisante.ch
https://www.unisante.ch/covid-fm

COVID-FM: Feuille de comptage journalière ASSISTANT·E MEDICAL·E

RAPPEL : **Ne comptabilisez pas les appels transférés à la ou au médecin**

Date 08 / 03 / 2021		Initiales / Identifiant poste de travail DA	
**Appels téléphoniques**		*Comptage*	**Total**
A	**Nombre total de téléphones non référés** au médecin (avec ou sans COVID)	𝍸 𝍸 𝍸 𝍸 𝍸 𝍸 𝍸 𝍸 III	43
B	**Nombre total de patient·e·s ayant rempli les critères de l'OFS d'une nouvelle suspicion de COVID-19**, dont …	𝍸 𝍸 𝍸 II	17
B1	… Nombre référés pour un test hors cabinet	III	3
B2	… Nombre de patient·e·s pour lesquels un rendez-vous a été pris pour un test en cabinet		

* Critères cliniques de suspicion COVID-19 de l'OFSP, valables au 15.03.2021 :
• Symptômes d'affection aiguë des voies respiratoires (p. ex. toux, maux de gorge, difficultés respiratoires, douleurs thoraciques) et/ou
• Fièvre sans autre étiologie et/ou
• Apparition soudaine d'une anosmie (perte de l'odorat) et/ou d'une agueusie (perte du goût) et/ou
• État confusionnel aigu ou détérioration de l'état de santé chez une personne âgée sans autre étiologie
• Autres symptômes moins spécifiques ou plus rares : douleurs musculaires, maux de tête, sensation de fatigue généralisée, rhume, symptômes gastro-intestinaux (p. ex. nausées, vomissements, diarrhées, douleurs abdominales), éruptions cutanées (p. ex. pseudo-engelures, exanthèmes urticariens, vésiculaires ou morbilliformes)

Lien vers les mises à jour: https://www.bag.admin.ch/dam/bag/fr/dokumente/mt/msys/covid-19-verdachts-meldekriterien.pdf.download.pdf/Criteres_de_suspicion_de_prelevement_d-echantillons_et_de_declaration.pdf

20.04.2021

Données saisies ☐
